# Computational Model for Membrane Transporters. Potential Implications for Cancer

**DOI:** 10.3389/fcell.2021.642665

**Published:** 2021-02-22

**Authors:** María Florencia Carusela, J. Miguel Rubi

**Affiliations:** ^1^Instituto de Ciencias, Universidad Nacional de General Sarmiento, Buenos Aires, Argentina; ^2^National Scientific and Technical Research Council, Buenos Aires, Argentina; ^3^Departament de Física de la Matèria Condensada, Universitat de Barcelona, Barcelona, Spain

**Keywords:** cancer transporters, membrane transport mechanisms, entropic forces, Langevin equation, cancer therapies

## Abstract

To explain the increased transport of nutrients and metabolites and to control the movement of drug molecules through the transporters to the cancer cells, it is important to understand the exact mechanism of their structure and activity, as well as their biological and physical characteristics. We propose a computational model that reproduces the functionality of membrane transporters by quantifying the flow of substrates through the cell membrane. The model identifies the force induced by conformational changes of the transporter due to hydrolysis of ATP, in ABC transporters, or by an electrochemical gradient of ions, in secondary transporters. The transport rate is computed by averaging the velocity generated by the force along the paths followed by the substrates. The results obtained are in accordance with the experiments. The model provides an overall framework for analyzing the membrane transport proteins that regulate the flows of ions, nutrients and other molecules across the cell membranes, and their activities.

## 1. Introduction

Cancer cells synthesize increased amount of fatty acids and protein building blocks to successfully divide and metastasize. To support their metabolism cancer cells require increased supply of metabolic substrates and nutrients. Plasma membrane transporters secure import of a wide range of substrates into the cytoplasm. Consistently, increased expression of several transporting proteins has been correlated to the increased metabolic activity of cancer cells and poor disease prognosis (Natecz, 2020; Sampedro-Núñez et al., 2020; Xu et al., 2020; Yamada et al., 2020).

There are two types of transporters, ATP biding cassette (ABC) and secondary active transporters. Secondary active transporters carry substrates across the plasma membrane using electrochemical gradient of ions. They bind the substrate on one side of the membrane followed by a conformational change allowing to release the substrate on the other side of the membrane (Boudker and Verdon, 2010). Several of these transporters have been shown to play an important role in cancer cells by increasing uptake of such substrates as glucose, glutamine, lactate, etc., and supporting cancer cell metabolism (Payen et al., 2017; Reckzeh et al., 2019; Scalise et al., 2020).

ABC transporters belong to a large family of transporters carrying several different substrates across the plasma membrane. To translocate substrates ABC transporters use the energy from ATP hydrolysis. They play an important role in keeping cellular homeostasis by regulating the level of several molecules including peptides, lipids, amino acids, or drugs (El-Awady et al., 2017; Neumann et al., 2017). Importantly, ABC transporters have also been shown to contribute to anti-cancer drug resistance presenting a major problem in finding an efficient anti-cancer therapy (Robey et al., 2018; Bock et al., 2019; Asif et al., 2020). These transporters carry out anti-cancer drugs efflux from cancer cells significantly decreasing the efficiency of anti-cancer therapies.

To be able to successfully overcome the problem of the increased transport of nutrients and metabolites and control drugs influx/efflux through transporters in cancer cells it is important to understand the exact mechanism of their structure and activity as well as their biological and physical features. This has been the objective of this article.

We have proposed a model that describes the trans-membrane transport and calculates the flow of substrates through the membrane. The model assumes that changes in transporter conformations induce forces that contribute to the translocation of substrates. Since entropy is a measure of conformations, the forces are referred to as entropic forces. These forces thus encode the information of the change in the available space of the substrates due to conformation changes (Zwanzig, 1992; Reguera and Rubí, 2001; Vazquez et al., 2008; Carusela and Rubi, 2017, 2018; Rubi, 2019). The computed translocation rates scale with the ratio between the time it takes for conformations to change and the time in which substrates diffuse through the transporter. This scaling behavior makes our model a general tool in the study of membrane transport.

## 2. The Model

**Figure 1:**
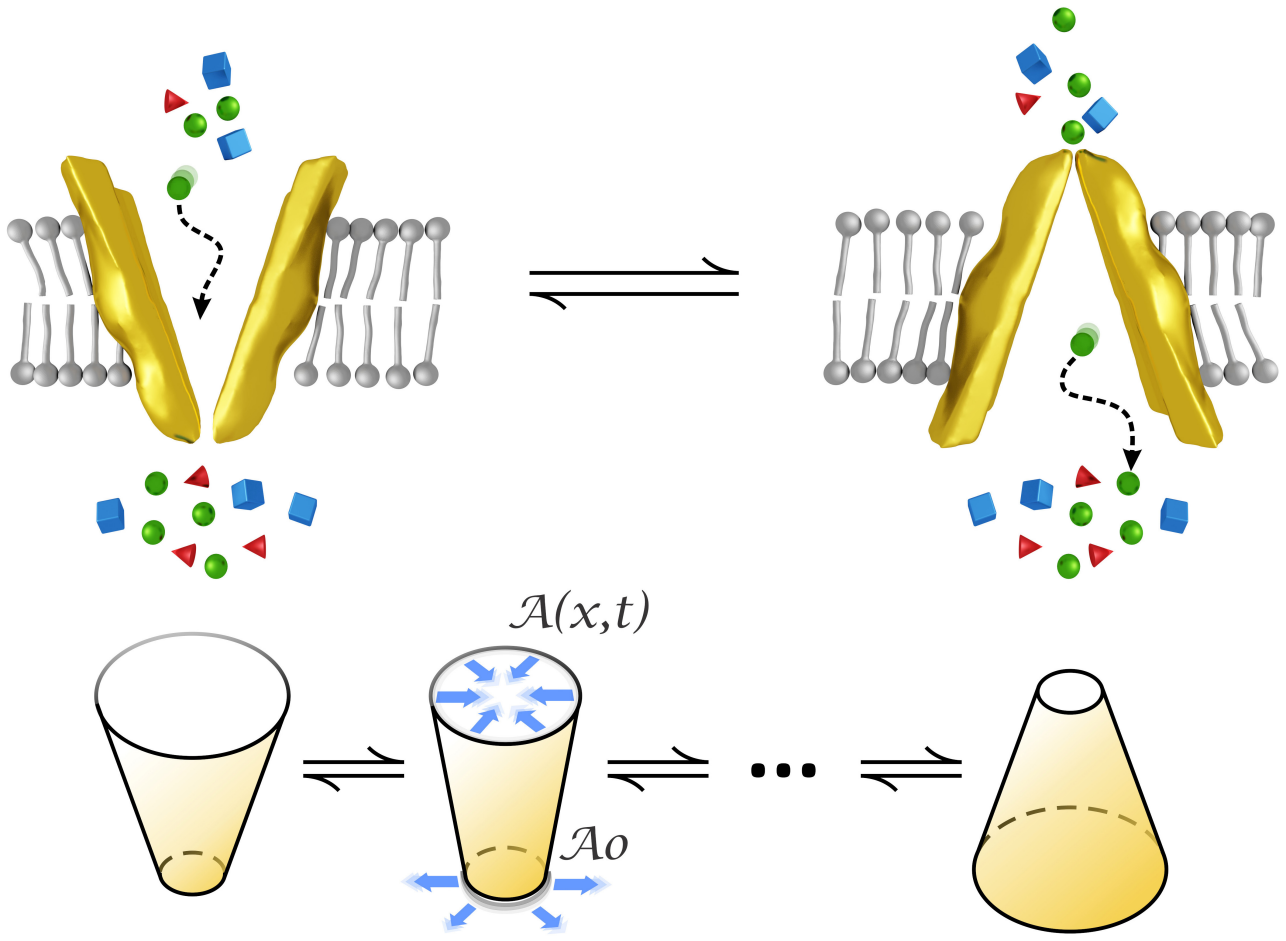
Conformational changes of a transporter modeled by an oscillating conical-shaped channel which closes/opens to the intra/extra cellular medium. $A_{0}$ stands for the cross-sectional area at the narrowest part whereas A(x,t) denotes that area at positions inside the transporter and time. Figures at the bottom represent the initial and final states and an intermediate state of the cone oscillations in the model.

We model a transporter as a small funnel-shaped motor whose structure changes over time by alternately opening and closing to the intra/extracellular medium, as represented in Figure 1. This form has been observed for example in P-glycoprotein whose structure is narrow at the cytoplasmic side, of about 9–25 Å in the middle, and wider at the extracellular surface (Loo and Clarke, 2001). The conical form has also been observed in pumps (Rubi et al., 2017) by means of crystallization experiments (Olesen et al., 2007).

This periodic movement makes it possible for substrates, such as aminoacids, ions, neurotransmitters, nutrients and different drugs, to overcome the potential barriers generated by interactions allowing them to pass to the other side of the plasma membrane.

Alternating gating increases transport efficiency with respect to that of diffusion. Transport of substrates through the membrane is the result of changes in its conformation. Primary active transporters couple substrate movements to a source of chemical energy, such as ATP hydrolysis. Secondary active transporters are driven by electrochemical gradients of ions. Transporters differ from ion channels in that their turnover rate is much slower than that of channels which is typically of the order of 10^6^*s*^−1^. The rate of ABC transporters such as LeuT, MsbA, and of MFS secondary transporters frequently falls within the range (10^−1^ − 10^3^)*s*^−1^ (Ashcroft et al., 2009; Liu et al., 2018; Fitzgerald et al., 2019).

### 2.1. Force Induced by Transporter Conformation Changes

**Figure 2:**
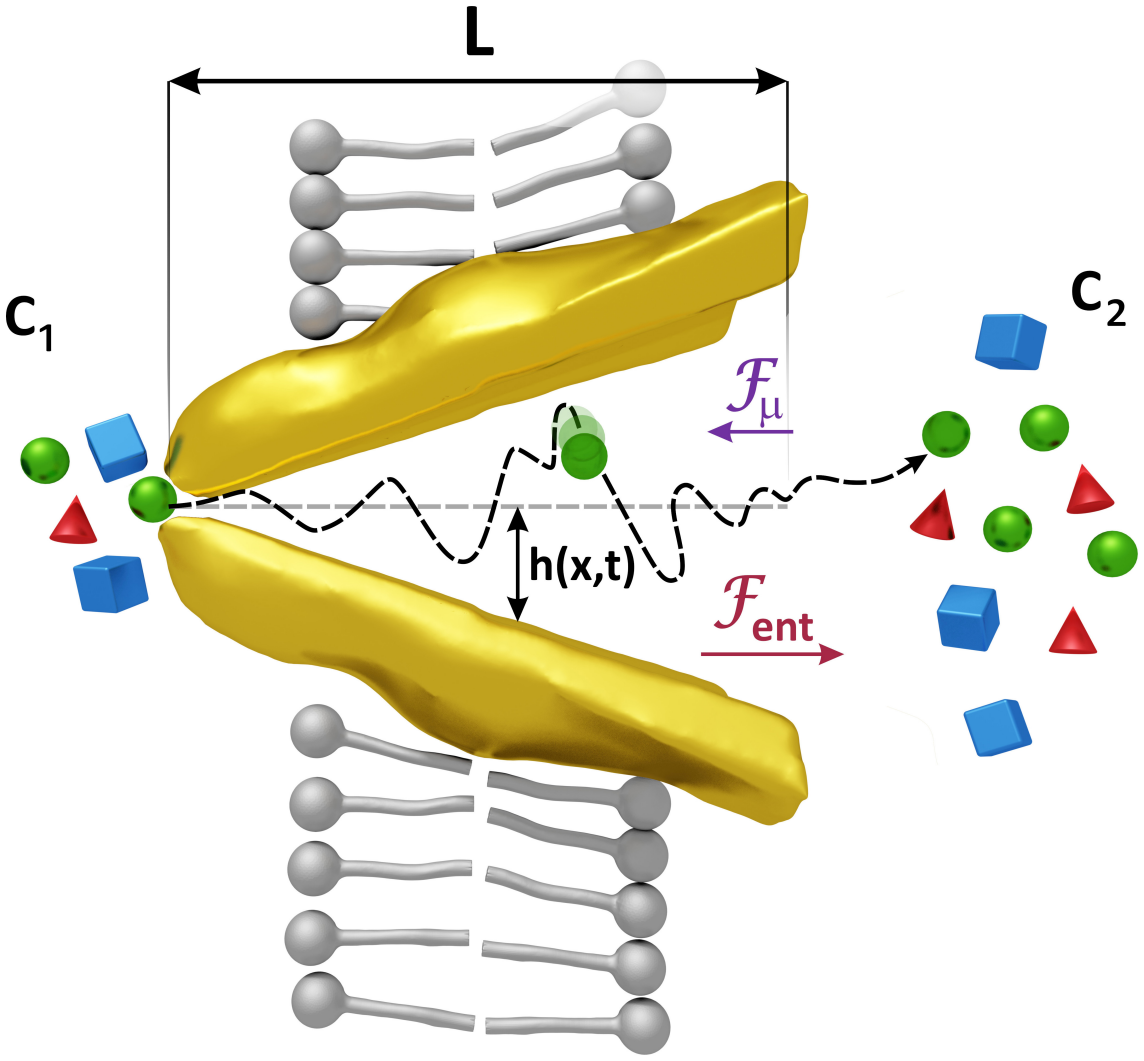
Forces acting on the substrates. Changes in the shape of the transporter induce an entropic force *F*_*ent*_, larger than the force *F*_μ_ generated by a difference of the concentrations *c*_1_ and *c*_2_, which helps to expel the substrates when the transporter is opened, and prevents the passage of them when it is closed. Substrates are also affected by the random motion of the molecules of the solvent whose average kinetic energy is proportional to *k*_*B*_*T*. The associated force has been denoted by *F*_*r*_ in Equation (1). The radius of the transporter *h*(*x, t*) = (*h*_*max*_ − *h*_*min*_)(*x*/*L* − 1/2) sin ω*t* + (*h*_*max*_ + *h*_*min*_)/2 varies with position and time reaching maximum and minimum values, *h*_*max*_ and *h*_*min*_, respectively. *x* = 0 is located at one extreme of the transporter.

Our model considers that the changes in the conformation of both types of transporters that allow the passage of the substrates entail a variation in the space they have to move. This fact affects the entropy of the substrates as this quantity measures the degree of disorder of a system which in our case is less in the narrow area of the transporter, where the substrates have fewer positions to occupy, and more in the wider area where the space available is greater. This difference of entropies between the narrow and the wide part of the transporter gives rise to a gradient of free energy and consequently to a force on the substrates that we will call entropic force $F_{ent}$ (see Figure 2).

The force arising from the uneven shape of the transporter must therefore be proportional to Δ*A* = *A*_2_ − *A*_1_, with *A*_1_ and *A*_2_ the cross-sectional areas at the entrance and at the exit of the transporter (see Figure 2) which depend on time. Studies on ion translocation in *Ca*^2+^-ATPase and in *Na*^+^/*K*^+^-ATPase have revealed that structural changes in a protein channel and their induced entropic forces contribute significantly to the transport of the ions (Rubi et al., 2017).

Changes in the concentration, or equivalently, in the chemical potential of the substrates on both sides of the plasma membrane generate a mass flow from high to low concentrations. The force associated with this effect, proportional to Δ*c* = *c*_2_ − *c*_1_, with *c*_1_ and *c*_2_ the concentrations at the entrance and at the exit of the transporter (see Figure 2), is lower than the entropic force and is directed in the opposite direction, so the net effect is a flow of substrates against the gradient, an active transport due to the catalyst effect of the transporter.

Substrates are also affected by the thermal motion of the solvent which exert a random force $F_{r}$ on them. The thermal energy is *k*_*B*_*T* and the thermal random force is given by $F_{r}=\sqrt{2k_{B}T\gamma }\eta \left(t\right)$, with η(*t*) a Gaussian random quantity of mean zero and correlation < η(*t*)η(*t*′)>=δ(*t*−*t*′), T the temperature, *k*_*B*_ the Boltzmann constant and $\gamma =\frac{k_{B}T}{D}$ the friction coefficient which is the inverse of the diffusion coefficient D in *k*_*B*_*T* units (Gardiner, 2004).

The previous forces acting on the substrates capture the essential factors involved in the translocation process. In the model, we assume that the resulting velocity *v* is given through the Langevin equation (Gardiner, 2004)

(1) $$\begin{array}{l} \gamma v=F_{ent}+F_{\mu }+F_{r} \end{array}$$

It has been shown that entropic forces are given by $F_{ent}=k_{B}T\frac{\nabla A\left(x,t\right)}{A\left(x,t\right)}$ (de Groot et al., 1963; Zwanzig, 1992; Reguera and Rubí, 2001; Kalinay and Percus, 2006; Vazquez et al., 2008; Rubi, 2019) and $F_{\mu }=-k_{B}T\frac{\nabla c\left(x\right)}{c\left(x\right)}$. In Equation (1), these forces act on the substrates at their time-dependent positions. The entropic force depends on the local radius h(x,t) of the channel (Reguera and Rubí, 2001) and has the direction of the cross-sectional area gradient, i.e., it contributes to expel the substrates, whereas the diffusive force has the opposite sign of the concentration gradient. A similar Langevin equation was proposed to model particle translocation through microfluidic channels promoted by an oscillating external potential (Tan et al., 2017).

To study how changes in conformation of the transporter affect the velocity of the substrate, we must model its geometry. A simple yet representative form is that of a conical shaped region of length L that oscillates periodically in time with a frequency ω (Carusela and Rubi, 2017, 2018), opening and closing to the intra/extra cellular environment, as sketched in Figure 2. The radius *h*(*x, t*) of the channel changes from a maximum value *h*_*max*_ to a minimum value *h*_*min*_ evolving in time as

(2) $$\begin{array}{l} h\left(x,t\right)=\left(h_{max}-h_{min}\right)\left(x/L-1/2\right)\sin\omega t+\left(h_{max}+h_{min}\right)/2 \end{array}$$

The value *x* = 0 is located at one extreme of the transporter.

### 2.2. Computation Protocol

Measurements of translocation rates are performed over a time interval long enough to comprise many time periods $\bar{T}$ of the conformational change cycle of transporters of the same kind. To obtain a representative value of the velocity V of the substrates, we must thus average the instantaneous velocity *v*(*t*), given in Equation (1), in time and over an ensemble of identical transporters. The average velocity is thus obtained as

(3) $$\begin{array}{l} V=\frac{1}{\bar{T}}\int_{\bar{T}}<v\left(t\right)>dt \end{array}$$

where < ... > means average over an ensemble of realizations or initial states of the system.

To calculate < *v*(*t*) >, we integrate Equation (1) using a stochastic Velocity-Verlet algorithm (Gränbech-Jensen and Farago, 2013). The computation method runs as follows. We consider a particle at the entrance of the channel, on the extracellular environment, and set its initial velocity according to a Boltzmann distribution, at *T* = 300*K*. We then calculate its local velocity according to Equation (1). Once the particle reaches the exit of the transporter into the intracellular medium, we calculate the time average $\frac{1}{\bar{T}}\int_{\bar{T}}dtv\left(t\right)$, assuming that when the particle leaves the channel it cannot re-enter. This protocol is repeated for a large set of initial particle conditions at the transporter entrance. The average (< .. >) of the obtained results gives us the value of V. In the model, we have considered that the force due to the concentration gradient in Equation (1) is practically constant along the transporter which means that $F_{\mu }\approx \frac{k_{B}T}{L}\frac{\Delta c}{\bar{c}}:=\frac{k_{B}T}{L}f_{\mu }$. The ratio $\frac{\Delta c}{\bar{c}}$ and therefore *f*_μ_ typically takes values in the range (10^−1^ − 1) (Sperelakis, 2000; Tashiro et al., 2005; Chu et al., 2013). The value of the entropic forces falls in the interval (1 − 10^1^), therefore $F_{\mu }<F_{ent}$ which means that changes in conformation is the main mechanism that regulates transport.

## 3. Results

**Figure 3:**
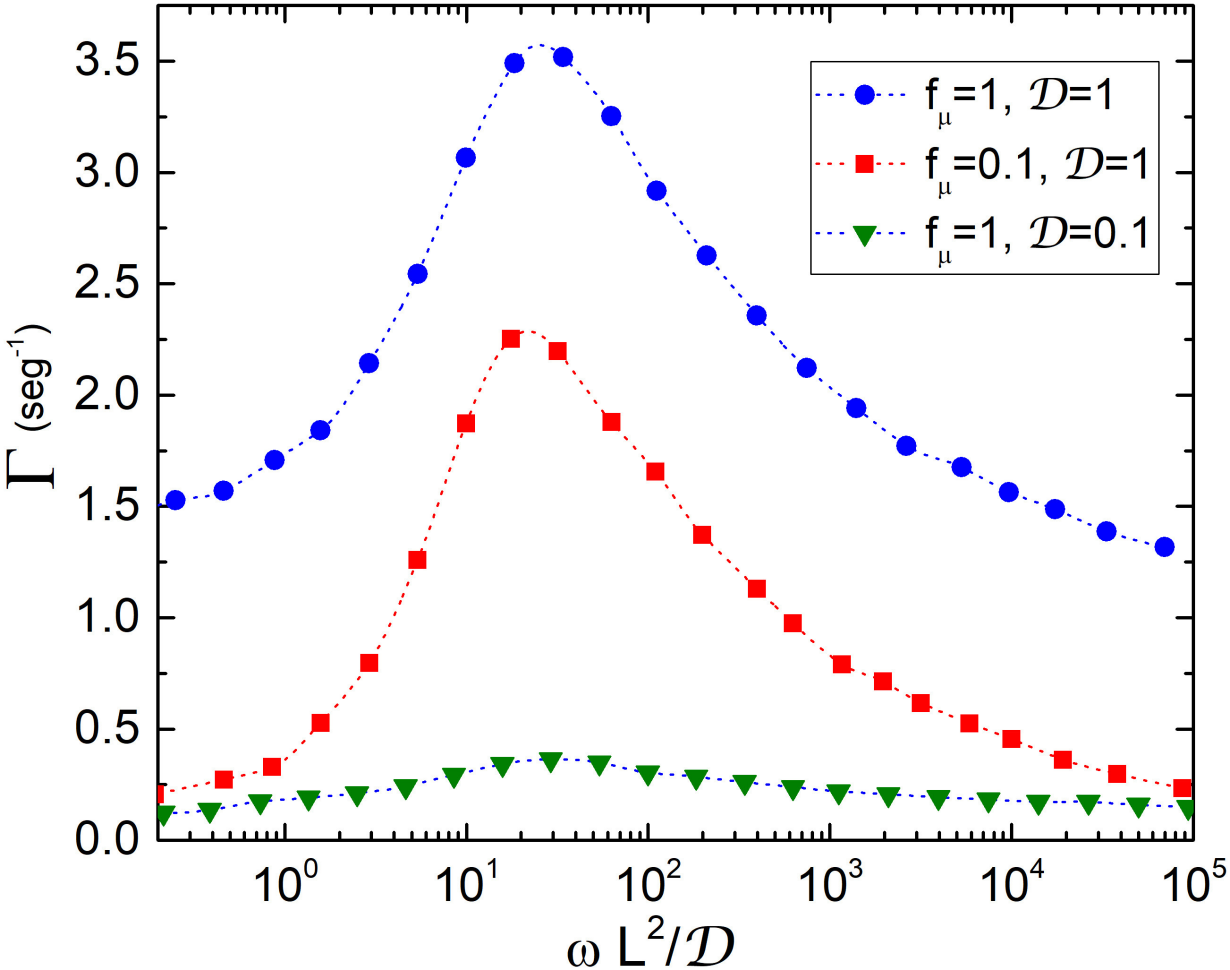
Transport rate Γ vs. ratio between the time conformations take to change and the diffusion time: $\omega L^{2}/D$. The quantities D, *f*_μ_, *L*, and ω which are defined in the text, have been measured in units of $D_{0}=10^{-2}\mu m^{2}/seg$, *k*_*B*_*T*/*L*_0_ (*T* = 300 K), *L*_0_ = 10*nm* and $L_{0}^{2}/D_{0}$, respectively. An enhancement of the velocity is observed at $\frac{\omega L^{2}}{D}\sim 30$ regardless of the values of *f*_μ_, thus showing that transporters work in an optimal scenario dominated by entropic forces induced by conformational changes.

Following the protocol described in the previous section, we compute the transport rate Γ=V/L which is plotted in Figure 3 vs. $\omega L^{2}/D$. This quantity represents the ratio between the time in which the conformation of the transporter changes and the time that substrates take to diffuse through the transporter. The presence of resonant peaks at $\frac{\omega L^{2}}{D}\sim 30$, practically independent of the values of *f*_μ_, reveals the occurrence of an amplification of the velocity of the substrates at a certain value of the oscillation frequency and therefore shows that transporters work under optimal transport conditions. The peaks become more pronounced when *f*_μ_ increases or equivalently the rates are higher when the concentration of substrates increases, as observed for example in experiments for LeuT transporters (Fitzgerald et al., 2019). The existence of an optimal frequency for transport of particles in microfluidic devices subjected to an external oscillatory potential was also found in Tan et al. (2017).

Considering transporters with length of the order of *L*_0_ = 10*nm* and typical diffusion coefficients for membrane proteins of Eukaryotic cells, $D_{0}=\left(10^{-2}-1\right)\mu m^{2}/seg$ (Kaňa, 2013), we obtain values for Γ in the range of (10^−1^ − 10^3^)*seg*^−1^. Our model also provides values of the rates in ion channels. In this case, one would expect that since particles are lighter they move faster. Typical values of $D_{0}$ for ions are in the range (10^3^ − 10^4^)μ*m*^2^/*seg*, therefore Γ takes values between (10^5^ − 10^6^)*seg*^−1^. These model predictions are in good agreement with data found in the literature for transport rates of slow and fast protein channels in membranes (Ashcroft et al., 2009). At the maximum opening configuration of the transporter, the entropic force changes from a value of the order of *f*_μ_ at the widest part, to about 20*f*_μ_ at the narrowness. Our results then show that transporters work in a regime dominated by entropic forces induced by conformational changes.

## 4. Conclusions

Membrane proteins transporters regulate the efflux/influx of substrates across plasma membrane of cancer cells and play a paramount role in the efficiency of cancer therapies as they regulate the retention of anticancer drugs in the cells. It is believed that the effectiveness of chemotherapy may be largely dependent on the activity of transporters. Knowing which is the intimate mechanism that makes possible the passage of substrates through the cell membrane is therefore a matter of vital importance.

In this article, we have proposed a computational model that analyses the activity of transporters. The model shows that changes in the transport configuration produced by the energy of ATP hydrolysis (ABC transporters) or by a electrochemical gradient of ions (secondary transporters) give rise to a force that makes the substrates able to overcome the potential barrier generated by the constrictions in order to pass to the other side of the membrane. Such a force results from the variation of the space that the substrates have to move within the transporter when its configuration changes, which generates an entropy gradient.

Transport rates are computed by following a simulation protocol in which we first obtain the time average of the velocity of the substrates which is subsequently averaged over a set of initial conditions of the position of a substrate. Our model reproduces experimental values of the rates for different ABC and secondary transporters and shows that they depend on substrate concentration, in accordance with data reported in recent experiments (Fitzgerald et al., 2019). It can also be used to compute translocation rates in ion channels showing that they are greater than for transporters, as observed in the experiments (Ashcroft et al., 2009). We have shown that the entropic force is greater than that produced by a mere concentration gradient and is directed in the opposite direction which shows that transport is active.

How readily substrates cross the membrane depends on the frequency of oscillation ω, which regulates the entropic force and therefore the changes in the conformation of the transporter, on the size of the substrates which is involved in D and on the size of the transporter *L*. The rates found scale with the combination of these quantities: $\omega L^{2}/D$ which represents the ratio between the time in which conformations change and the time it takes for the substrates to diffuse. Our results could therefore be applied in general to membrane transport, to transport in microfluidic devices and peristaltic channels and to capture cellular heterogeneity beyond cancer cells (Pu et al., 2016; Łapińska et al., 2019; Rhia et al., 2020). Monitoring the entropic force by means of drugs could help to regulate transport activity leading to the design of better transport modulators that can be used in anti-cancer therapies.

## Data Availability Statement

The raw data supporting the conclusions of this article will be made available by the authors, without undue reservation.

## Author Contributions

All authors have made a substantial contribution to the work and approved it for publication.

## Conflict of Interest

The authors declare that the research was conducted in the absence of any commercial or financial relationships that could be construed as a potential conflict of interest.
